# Successful treatment of idiopathic multicentric Castleman disease with TAFRO and kidney involvement: case report and literature review

**DOI:** 10.1080/0886022X.2026.2671463

**Published:** 2026-06-17

**Authors:** Ahmed I. Kamal, Hima Doppalapudi, Thien Ho, Salem Vilayet, Vishu Pasham, Milos Budisavljevic, Anand Achanti

**Affiliations:** ^a^ Internal Medicine Department, Nephrology Division, Medical University of South Carolina, Charleston, SC, USA; ^b^ Mercy Medical Center, Cedar Rapids, IA, USA; ^c^ Pathology and Laboratory Medicine Department, Medical University of South Carolina, Charleston, SC, USA

**Keywords:** iMCD-TAFRO, IL-6, siltuximab, kidney disease, kidney histopathology

## Abstract

Idiopathic multicentric Castleman disease (iMCD) is a rare hematological disease of heterogenous presentation, with symptoms ranging from mild to severe and life-threatening. A severe subtype of iMCD is characterized by thrombocytopenia, anasarca (including pleural effusion and ascites), fever, reticulin myelofibrosis or renal dysfunction, and organomegaly (iMCD-TAFRO). Early identification and treatment of iMCD-TAFRO is crucial because of its rapid onset, severe renal dysfunction and high mortality risk. However, diagnosing iMCD-TAFRO can be difficult due to a lack of diagnostic biomarkers and symptoms that overlap with a variety of lymphoproliferative, infectious, and rheumatological diseases. Although the exact etiology of iMCD-TAFRO is unknown, interleukin (IL)-6 has been identified as a critical pathological driver, and the IL-6 antagonist siltuximab is recommended as first-line treatment. While many patients with iMCD-TAFRO have abnormal kidney function and will require nephrology care, evidence describing kidney histopathology and kidney involvement in this condition is limited. Herein, we review the disease state and recent advances with a focus on renal histopathology and the mechanisms of kidney injury in iMCD-TAFRO. We also present and discuss a case report of a patient with iMCD-TAFRO and acute kidney failure. Our aim is to draw attention to the spectrum of kidney involvement in iMCD-TAFRO, as well as raise awareness to the importance of early recognition and appropriate treatment of this rare condition.

## KEY MESSAGES

What is known: Many patients with iMCD-TAFRO have abnormal kidney function and may require nephrology care and dialysis in some cases, but evidence describing kidney histopathology and kidney involvement in this rare condition is limited. Published literature to date are mostly focused on reports of membranoproliferative glomerulonephritis and thrombotic microangiopathy on kidney biopsy.

This study adds: We provide an in-depth review of recent advances on iMCD-TAFRO focusing on renal histopathology and the mechanisms of kidney injury. We also present and discuss the kidney findings and treatment outcomes of a patient with iMCD-TAFRO and acute kidney failure.

Potential impact: Our report draws attention to the spectrum of kidney involvement in iMCD-TAFRO and raises awareness of the importance of early recognition and appropriate treatment of this rare condition. The data in this report will be of interest to nephrologists who may encounter patients with iMCD-TAFRO and acute kidney injury.

## 1. Introduction

Castleman disease (CD) describes a group of rare hematological disorders with similar histopathology but varying etiologies and clinical manifestations [1–3]. This condition is classified based on lymph node involvement as either unicentric CD (UCD, which involves a single lymph node or lymph node station) or multicentric CD (MCD, with ≥2 lymph node stations involved), though recently an intermediate regional/oligocentric CD phenotype (with 2–3 adjacent lymph node stations involved and a clinical course similar to UCD) has been reported [4]. MCD is subdivided by etiology as human herpes virus type 8 (HHV-8)-associated MCD; polyneuropathy, organomegaly, endocrinopathy, monoclonal plasma cell disorder, skin changes (POEMS)-associated MCD; and idiopathic MCD (iMCD) [1]. Patients with iMCD can present with an indolent subtype called idiopathic plasmacytic lymphadenopathy (iMCD-IPL) characterized by marked polyclonal hypergammaglobulinemia and plasmacytic histology, and some have a severe form characterized by thrombocytopenia, anasarca, fever, reticulin myelofibrosis, renal dysfunction, and organomegaly (iMCD-TAFRO) [5,6]. It should be noted that most patients do not meet criteria for iMCD-IPL or iMCD-TAFRO, and instead present with a mild phenotype called not otherwise specified (iMCD-NOS). The iMCD-TAFRO subtype has the most aggressive clinical course and poorest prognosis [7]. Many patients with iMCD-TAFRO present with acute kidney injury, requiring nephrology care and dialysis [8]. However, evidence describing classical kidney involvement in iMCD-TAFRO is limited, and additional information is needed to understand how to best diagnose and treat these patients. Herein, we review the current CD landscape and focus on the different degrees of kidney involvement in patients with iMCD-TAFRO. We also include a case report of a patient with iMCD-TAFRO and acute kidney disease to add to the growing evidence of abnormal kidney histological findings in patients with this rare condition.

## 2. Clinical presentation, pathology and severity

Castleman disease exists on a spectrum of lymph node involvement and severity. UCD typically presents as an enlarged mass in the mediastinum, which may be visually apparent or detected incidentally. Most patients are asymptomatic, but some may present with symptoms related to compression of nearby anatomical structures [1]. Laboratory abnormalities can include hypergammaglobulinemia, elevated erythrocyte sedimentation rate (ESR), and anemia [1]. Up to 90% of UCD cases present with hyaline vascular (HV) histopathology, which is characterized by atrophic germinal centers circumscribed with concentric rings of lymphocytes in an ‘onion-ring’ pattern and containing dysplastic follicular dendritic cells; the germinal centers are penetrated by hyalinized vessels giving a ‘lollipop’ appearance [9].

Unlike UCD, MCD is almost always symptomatic [10]. Patients present with multifocal lymphadenopathy and signs of systemic inflammation, most commonly fever, night sweats, fluid retention, and renal dysfunction [1,10–12]. These symptoms can be episodic, progressive, or chronic, and in severe cases can be fatal if left untreated [10,13]. Laboratory tests often reveal cytopenias, abnormal liver function tests, markedly elevated C-reactive protein (CRP) and ESR, and proteinuria. The histopathology of MCD is more varied than UCD; it may present with a hypervascular type (hyperV), the plasmacytic (PC) type, or a mixed type. The hyperV nomenclature is used to differentiate vascular MCD from HV UCD, though HV MCD and HyperV MCD are often used interchangeably by many authors. The lymph nodes in the PC type are characterized by hypertrophic germinal centers, with intrafollicular spaces populated by sheets of plasma cells and vascular proliferations; as its name suggests, the mixed type exhibit features of both the HyperV and PC types [1,13].

The etiologic agent varies for the different subtypes of MCD. Uncontrolled infection with HHV-8 is often the pathogenic driver of MCD, especially in patients who are immunocompromised due to infection with human immunodeficiency virus (HIV). In these HHV-8-associated MCD cases, CD20^+^ B cells act as reservoirs for HHV-8 infection and replication, with symptomology stemming from production of a viral homolog of IL-6 and subsequent downstream upregulation of proinflammatory human cytokines [1,14]. For POEMS-associated MCD, up to 60% of cases are caused by malignant monoclonal plasma cells, which drive reactive lymph node changes with CD features [13]. Cases of MCD which are not concurrent with HHV-8 infection or POEMS syndrome do not have a clear etiology and are considered idiopathic (iMCD). While the exact etiology remains unknown, IL-6 has been identified as a key pathological driver of iMCD [1]. Episodes of disease flare may be marked by mild lymphadenopathy and B-symptoms, or in severe cases present acutely with extensive inflammation, organomegaly and organ dysfunction, pleural effusion, anasarca, and ascites [7].

### 2.1. iMCD-NOS, iMCD-IPL, iMCD-TAFRO and TAFRO syndrome

Cases of iMCD in which thrombocytopenia, anasarca, renal dysfunction or reticulin fibrosis, fever, and organomegaly are present are classified as iMCD-TAFRO [3,7]. Ferritin and sCD25 are only modestly elevated in iMCD-TAFRO, whereas CRP is very high (often >100 mg/L) [15]. The gamma globulins are usually low, normal or modestly elevated in iMCD-TAFRO (5–15 g/L), in contrast to iMCD-IPL (usually >35 g/L) [6]. The cytokine profile, histopathology and clinical course of iMCD-TAFRO are different from iMCD-NOS; patients often have less markedly elevated serum IL-6 and increased serum vascular endothelial growth factor (VEGF), the lymph nodes are often highly vascularized and demonstrate the HyperV histopathology type, and the patients typically present with acute and severe disease. Additional differences include frequently elevated alkaline phosphatase (ALP) without hyperbilirubinemia or transaminase elevation, and normal to mildly elevated gammaglobulin [2,16].

Differentiating iMCD-TAFRO and TAFRO syndrome is challenging due to substantial overlap of signs and symptoms and lack of specific biomarkers. While the first descriptions of TAFRO syndrome by Takai and colleagues in 2010 focused on its hyperinflammation symptoms, acute onset, and rapid progression, current perspectives now acknowledge how TAFRO presents as a constellation of nonspecific symptoms that can occur concurrently with other conditions, including malignancies, rheumatologic disorders, infections, and POEMS syndrome [2,17,18].

Due to the heterogeneity in disease presentation, Nishimura et al. introduced the categories of probable iMCD-TAFRO and TAFRO syndrome without iMCD; these designations are based on the number of diagnostic criteria met (see below) [2]. There was an open debate as to whether TAFRO was exclusively a subtype of iMCD, a distinct clinical entity, or both [2,5, 7,19]. A recent retrospective analysis of data collected from 11 rheumatology and hematology centers in Japan identified 104 patients with TAFRO, of which 26 (25%) did not have lymphadenopathy, confirming that TAFRO can in fact exist without CD [20]. To date, there is no available data regarding the proportion of patients in the Western hemisphere who have TAFRO without CD. It is important to note that there is significant overlap in consensus diagnostic guidelines and treatment modalities for iMCD-TAFRO and TAFRO syndrome, and it has been proposed that iMCD-TAFRO and TAFRO without proven iMCD or other underlying pathologies be considered a single clinical entity [5,7].

### 2.2. Consensus diagnostic criteria for iMCD-TAFRO

Diagnosing iMCD-TAFRO can be challenging due to lack of specific diagnostic biomarkers and a heterogenous clinical presentation, requiring differential workup and collaboration across multidisciplinary teams. There are currently two diagnostic frameworks for TAFRO syndrome, which differ in their dependence on lymph node biopsy and TAFRO’s relation to iMCD (Table 1) [2,22]. Consensus criteria for iMCD-TAFRO require an excisional lymph node biopsy with histopathology consistent with the criteria listed in Table 1. As both criteria have significant overlap, and an excisional lymph node biopsy may not be feasible (due to patient critical condition, risk of bleeding, and/or inaccessible lymph node location), both criteria should be considered to diagnose iMCD-TAFRO [19]. When an excisional lymph node biopsy is not possible, a chest CT may be of diagnostic utility. Patients with TAFRO may have an apparent enlarged anterior mediastinum lacking a solid mass, described as ‘matted’, due to a mixture of adipose and soft tissues [23].

**Table 1.** Diagnostic criteria for iMCD-TAFRO and TAFRO Syndrome [1,21].

iMCD-TAFRO	TAFRO Syndrome
**Clinical criteria (all 4 required)** Thrombocytopenia Anasarca Fever (≥ 37.5ºC) or hyperinflammatory status (CRP ≥ 2.0 mg/dL) Organomegaly	**Major criteria (all 3 required)** Thrombocytopenia Anasarca Systemic inflammation defined as fever (≥ 37.5ºC) and/or CRP ≥ 2.0 mg/dL
**Pathological criteria (required)** Lymph node biopsy with histopathological features consistent with iMCD, which include atrophic germinal centers, concentric rings of mantle zone cells, and interfollicular hypervascularization or plasmacytosis	**Minor criteria (≥2 required)** CD-like features on lymph node biopsy Reticulin myelofibrosis and/or increased number of megakaryocytes in bone marrow Mild organomegaly, including hepatomegaly, splenomegaly and lymphadenopathy Progressive renal insufficiency
**Additional criteria (> 1 required)** Renal insufficiency (pre-treatment eGFR ≤ 60 mL/min/1.73 m^2^, creatinine >1.1 mg/dL [female]/ >1.3 mg/dL [male], or failure requiring dialysis) TAFRO-consistent bone marrow, with reticulin fibrosis or megakaryocyte hyperplasia	N/A
**Exclusion criteria (required)** Infectious disease (HHV-8, EBV-associated lymphoproliferative disorder, acute HIV infection, tuberculosis, COVID-19 cytokine storm syndrome) Autoimmune / rheumatologic disease (SLE, Sjogren’s syndrome, rheumatoid arthritis, adult-onset Still disease, juvenile idiopathic arthritis, IgG > 3,400 mg/dL [suggestive of autoimmune disease or plasma cell dyscrasias], primary hemophagocytic lymphohistiocytosis Malignancy (including but not limited to malignant lymphoma, multiple myeloma, metastatic cancer, POEMS syndrome)	**Exclusion criteria (required)** Malignancies, including lymphoma, myeloma, mesothelioma Autoimmune disorders, including SLE, Sjogren’s syndrome, ANCA-associated vasculitis Infectious disorder (including acid fast bacterial infection, rickettsial disease, Lyme disease, and severe fever with thrombocytopenia syndrome) POEMS syndrome Hepatic cirrhosis Thrombotic thrombocytopenic purpura/ hemolytic uremic syndrome
**Supportive criteria (not required)** Renal insufficiency (pre-treatment eGFR ≤ 60 mL/min/1.73 m^2^, creatinine >1.1 mg/dL [female]/ >1.3 mg/dL [male], or failure requiring dialysis) TAFRO-consistent bone marrow, with reticulin fibrosis or megakaryocyte hyperplasia Absence of polyclonal hypergammaglobulinemia (immunoglobulin G ≤ 1.2x ULN by nephelometry Elevated ALP with mild to no elevation in bilirubin and transaminases	**Additional criteria (not required)** TAFRO features: Elevated serum ALP Lymphadenopathy <1.5 cm in diameter Transudative pleural effusion and ascites with high IL-6 and VEGF levels (higher than serum levels) Hepatosplenomegaly usually mild and only confirmed by CT-scan Hypergammopathy (>3000 mg/dL) is rare in TAFRO. Obvious monoclonal protein should be absent. Elevated LDH may indicate lymphoma. Exclude intravascular large B cell lymphoma with bone marrow aspiration and random skin biopsy Exclusion criteria for CD and immune thrombocytopenia have not been determined Exclude autoimmune disorders with rheumatoid factor, anti-nuclear antibody, anti-SS-A/Ro antibody, MPO-ANCA, and PR3-ANCA Exclude mycobacterial infection (incl. tuberculosis) with interferon-gamma release assays and adenosine deaminase in pleural effusion

ANCA, antineutrophil cytoplasmic antibody; anti-SS-A/Ro, anti–Sjögren’s-syndrome-related antigen A autoantibodies; ALP, alkaline phosphatase; CD, Castleman disease; COVID19, Coronavirus disease 2019; CRP, C-reactive protein; CT, computed tomography; EBV, Epstein-Barr virus; eGFR, estimated glomerular filtration rate; HHV-8, human herpes virus-8; HIV, human immunodeficiency virus; IgG, immunoglobulin G; IL-6, interleukin-6; iMCD, idiopathic multicentric Castleman disease; LDH, lactate dehydrogenase; MPO-ANCA, myeloperoxidase antineutrophil cytoplasmatic antibody, POEMS, polyneuropathy, organomegaly, endocrinopathy, monoclonal plasma cell disorder, skin changes; PR3-ANCA, anti-proteinase 3-antineutrophil cytoplasmatic antibody: SLE, systemic lupus erythematosus; TAFRO, thrombocytopenia, anasarca, pleural effusions, fevers, reticulin fibrosis or renal dysfunction, and organomegaly; ULN, upper limit of normal; VEGF, vascular endothelial growth factor.

Additional criteria have been established which stratify iMCD by disease severity for the purpose of informing treatment [24]. Severe disease is defined as having at least two of the following: Eastern Cooperative Oncology Group Performance Status (ECOG-PS) >2, stage IV renal dysfunction, anasarca and/or ascites and/or pleural/pericardial effusion, hemoglobin <8 g/dL, or pulmonary involvement/interstitial pneumonitis with dyspnea.

### 2.3. Renal histopathology of iMCD-TAFRO

While progressive kidney failure or acute kidney injury are common in iMCD-TAFRO, evidence describing the kidney histopathology in this patient population is limited, as biopsy is often precluded by the critical nature of the disease and bleeding risk [19]. In a case series of 7 TAFRO patients who underwent renal biopsy, all cases displayed endothelial cell swelling and glomerular basement membrane double contouring, with mesangiolysis or mesangial loosening, consistent with a membranoproliferative glomerulonephritis (MPGN)-like pattern with endothelial injury [25]. All patients had acute kidney injuries at different stages and 57% of them needed kidney replacement therapy [25]. Using electron microscopy, investigators also reported endothelial cell swelling and loss of mesangial architecture, as well as loss of endothelial cell fenestrations. Another case report described MPGN in an iMCD-TAFRO patient, with an accompanying literature search identifying 19 iMCD-TAFRO patients with renal biopsies, of which 8 (42%) were described as MPGN-like and 11 (52%) were described as thrombotic microangiopathy (TMA)-like glomerulopathy, though without fibrin thrombi in glomerular vasculature [26]. In this report, the clinical, biological (including renal function and urinary sediment as well as IL-6 and VEGF levels), and outcome characteristics were similar between patients with MPGN and TMA-like presentation. The authors suggest there is a histopathological and likely a physiopathology continuum between TMA and MPGN, where TMA seems to appear first and gradually move toward an MPGN phenotype. Accordingly, the diagnosis may alternate based on how early the biopsies were performed. Additional review of the available literature reporting data from patients with iMCD-TAFRO/TAFRO syndrome and renal failure shows a histology pattern consistent with MPGN and TMA on kidney biopsy [21,27–29].

### 2.4. Mechanisms of kidney injury in iMCD-TAFRO

While IL-6 has been identified as a key pathological driver of iMCD, not all patients have elevated IL-6 and other factors may be involved. Whole lymph node transcriptome analysis in patients with iMCD showed upregulation of genes in the mammalian target of rapamycin (mTOR) complex, complement and coagulation cascade (*C4A* and *C4B*), as well as the B cell-attracting chemokine *CXCL13*, and the angiogenic mediator placental growth factor (*PGF*, a ligand of VEGF receptor 1 [VEGFR1]) [30]. These findings were generally well aligned with another transcriptomic analysis study focusing on immune-related genes, which reported overexpressed *CXCL13* and upregulated genes involved in the complement cascade, VEGF signaling, and mTOR pathway [31]. These studies highlight the importance of angiogenesis, consistent with the hypervascularity observed in iMCD-TAFRO, as well as the potential involvement of the complement cascade and mTOR pathway in pathogenesis. Of note, complement dysregulation is the predominant mechanism of endothelial injury in atypical hemolytic uremic syndrome (also known as complement-mediated TMA), while mTOR dysregulation has been linked with a variety of kidney disorders including acute kidney injury, kidney fibrosis, glomerular disease, and polycystic kidney disease [32,33]. These two mechanisms could potentially drive the kidney damage observed in patients with iMCD-TAFRO.

Additional analysis of the cytokine microenvironment found that IL-6 was overexpressed in interfollicular areas of affected lymph nodes in MCD (observed in 76% of cases) when compared to UCD (25%) and reactive non-CD controls (20%), particularly in CD31-positive vascular structures of endothelial or lymphatic origin [31]. To a lesser degree, all samples showed some IL-6 expression in B cell rich mantle zones and GCs. These findings suggest that vascular cells, rather than plasma cells, are the major source of excess IL-6 in this condition. This is corroborated by a recent report of the source of IL-6 production and its transcriptional regulation across multiple iMCD subtypes, which showed that vascular endothelial cells are the major source of IL-6 in samples collected from patients with iMCD-TAFRO and that elevated serum IL-6 in these patients is secondary to upregulated cytokine storm-related genes (e.g., TNFα, IL-1R, mTOR, and VEGFA) [34,35]. In contrast, plasma cells were identified as the major source of IL-6 in the IPL subtype. Overall, these data may provide additional insight into the mechanisms of kidney injury in iMCD-TAFRO. One hypothesis is that kidney injury may be the result of a negative feedback loop in which increased circulating VEGF and IL-6 lead to decreased glomerular VEGF and loss of endothelial fenestrations [36]. Another hypothesis is loss of glomerular permeability and resultant microvascular injury, in which TMA is driven by autoantibodies produced by clonal B cells, in tandem with increased serum VEGF, disrupting the glomerular VEGF gradient [37].

## 3. Treatment of iMCD-TAFRO

### 3.1. Consensus treatment guidelines

While the exact etiology of iMCD is unknown, consensus treatment guidelines focus on targeting the underlying hypercytokinemia. These guidelines do not distinguish iMCD-TAFRO from other subtypes of iMCD, but rather focus on different treatment strategies based on disease severity. There are two organizations with detailed consensus treatment guidelines for iMCD: The NCCN Clinical Practice Guidelines in Oncology (NCCN Guidelines^®^) and the International Consensus Treatment Guidelines developed by the Castleman Disease Collaborative Network (CDCN) [24,38]. The anti-IL-6 monoclonal antibody siltuximab is the sole therapy approved by the FDA to treat iMCD and is recommended as a first-line treatment option per the CDCN and NCCN Guidelines^®^ [24,38]. The approved dosage is 11 mg/kg intravenous (IV) infusion every 3 weeks. In addition, the consensus CDCN guidelines suggest that patients diagnosed with severe iMCD who have marked organ dysfunction, poor performance status, and require intensive critical care should receive weekly siltuximab infusions (11 mg/kg) for up to 4 weeks. Patients who respond to treatment should remain on the approved treatment schedule (11 mg/kg every 3 weeks) indefinitely with tapering of corticosteroids. Alternative therapies for patients who do not respond to siltuximab include tocilizumab (IL-6 receptor antagonist), rituximab (anti-CD-20 monoclonal antibody), and/or cytotoxic chemotherapy. Corticosteroids can also be used as adjunctive therapy to anti-IL-6 agents or rituximab. If patients with severe disease do not show any response to siltuximab within 1 week or show any signs of deterioration, aggressive combination chemotherapy should be considered immediately [24]. In a case series/report of patients with iMCD-TAFRO who were refractory to IL-6 blockade, the mTOR inhibitor sirolimus and the TNFα inhibitor adalimumab, respectively, have been shown to induce durable remission and may be considered as alternative treatment options for these patients [39,40].

There are currently no consensus treatment guidelines for TAFRO syndrome outside of the context of iMCD, and no conclusive evidence from randomized, controlled clinical trials to assess treatment efficacy [19]. A treatment strategy for TAFRO syndrome as a separate entity from MCD was first proposed by Masaki et al. in 2015; however, treatment guidelines are based on case reports, analyses of registry data, expert opinion, and physician experience [41]. TAFRO is treated empirically with high-dose corticosteroids, blockade of IL-6 signaling with the monoclonal antibodies siltuximab or tocilizumab, or other options such as rituximab, calcineurin inhibitors, and cytotoxic chemotherapy; these modalities can be utilized as monotherapy or as part of a combination therapy approach.

## 4. Successful treatment of severe iMCD-TAFRO with siltuximab

### 4.1. Case report

A 31-year-old white female at 16 weeks gestation with her first pregnancy presented with chest pain, fever, and dyspnea for one week. Her medical and surgical history were unremarkable. On exam, the patient had poor inspiratory effort, bilateral wheezing and rales, and required supplemental oxygen. The patient also had moderate to severe pitting edema along with pronounced cervical bilateral lymphadenopathy. D-dimer was elevated, but there was no radiographic evidence of pulmonary embolism, and screening for upper respiratory tract infections was negative. Bloodwork showed elevated white blood cell (WBC) count (23 x 10^9^/L), elevated CRP (22.5 mg/dL), low hemoglobin (8.5 g/dL), low platelets (118 x 10^9^/L), and serum creatinine (sCr) of 1.1 mg/mL. Fetal heart sounds were normal prior to hospitalization; however, fetal demise was diagnosed on admission. Volume overload in the patient was treated with albumin and furosemide, but she did not respond (sCr 2.2 mg/dL on day 4) and became anuric. The patient’s respiratory status worsened and required mechanical ventilation. Follow-up assessments showed worsening thrombocytopenia (31 x 10^9^/L) with further elevation of CRP (27.5 mg/dL) and sCr (3.7 mg/dL), and no improvements of hypervolemia, anuria, and metabolic derangements. Due to the decline of her condition, the patient was placed on kidney replacement therapy, which improved her volume status and allowed her to be extubated.

**Figure 1.**
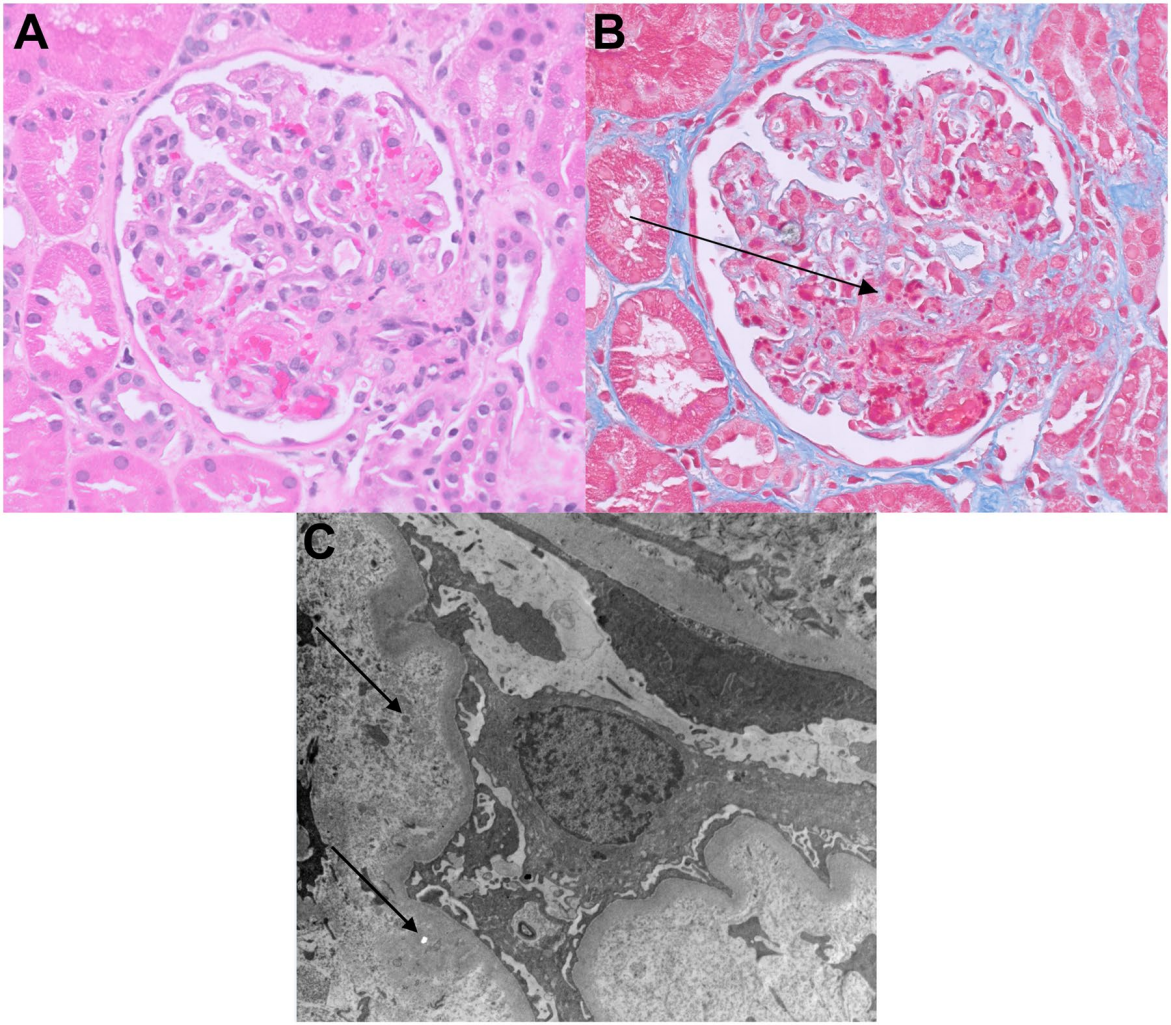
H & E (A) and trichrome (B) stains illustrating RBC fragmentation within the glomerular tufts, and RBC fragments percolating through the mesangium (denoted by arrow in 1B, 400x). Electron microscopy (C) showing loss of endothelial fenestrations and swelling of the endothelial cells, with an expansion of the subendothelial space by electron-lucent material (black arrows), compatible with an acute presentation of TMA (6000x). RBC, red blood cell; TMA, thrombotic microangiopathy.

A kidney biopsy showed membranoproliferative glomerulonephritis (MPGN) with TMA and red blood cell (RBC) fragmentation (Figure 1A,B). Electron microscopy analysis of the kidney tissue showed endothelial cell swelling, loss of endothelial cell fenestrations, and expansion of the subendothelial space, confirming an acute presentation of TMA (Figure 1C).

**Figure 2.**
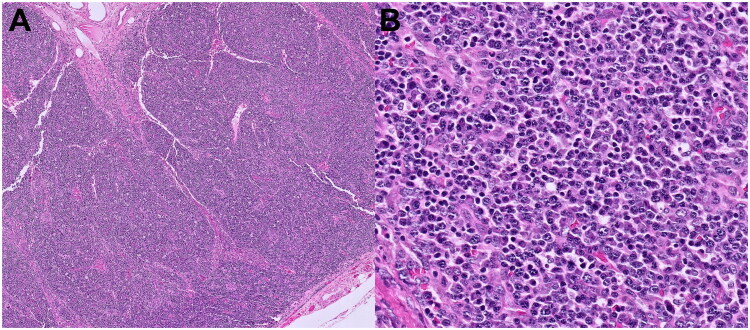
(A) Representative histopathology from a supraclavicular lymph node sample shows effacement of the lymph node without any obvious germinal centers and atretic follicles (H&E-stained section, 5x magnification). (B) Higher power magnification shows increased vascularity and marked plasmacytosis; most of the cellularity within the lymph node to be composed of polytypic, non-light chain restricted plasma cells (H&E-stained section, 40x magnification).

In view of the early complex presentation, we had a wide differential diagnosis including disseminated intravascular coagulation (DIC), viral infection, atypical hemolytic uremic syndrome (aHUS) and secondary TMA. Additional diagnoses also were considered for work-up including hemolysis, elevated liver enzymes and low platelets (HELLP) syndrome, gestational thrombocytopenia, autoimmune diseases and thrombotic thrombocytopenic purpura (TTP). Viral screening was negative for HHV-8, HHV-6, Epstein-Barr virus (EBV), cytomegalovirus (CMV), and HIV, and there were no findings on rheumatology and immunology work-up. Additional screenings were negative for cytoplasmatic and perinuclear anti-neutrophil cytoplasmic antibody (C-ANCA, p-ANCA), anti-nuclear antibodies (ANA), anti-glomerular basement membrane antibody (anti-GBM), lactate dehydrogenase (LDH), international normalized ratio (INR), hepatitis panel, anticardiolipin, beta-2 glycoprotein, antistreptolysin O (ASO), anti-double-stranded DNA (dsDNA), as well as a gastrointestinal panel. Moreover, aHUS genetic panel was unremarkable and flow cytometric analysis for acute leukemia was also negative. TTP was not likely as the ADAMTS 13 activity was 41%. Head and neck ultrasound showed multiple enlarged lymph nodes, and affected tissue was first collected by fine needle aspiration (yielded insufficient sample for analysis) followed by excisional biopsy of the supraclavicular lymph node (Figure 2). CD20 and CD79a markers for B-cells showed diminished follicular architecture/attenuation. The follicular architecture was retained, with CD21/CD23 staining showing an intact FDC meshwork. The features were overall compatible with follicular regression. Furthermore, a panel of CD138 stains and Kappa/Lambda *in situ* hybridization showed numerous interfollicular plasma cells that were associated with increased vascularity. EBV and HHV8 stains were negative within the biopsy. Flow cytometry did not detect any abnormal B-cell, T-cell, or plasma cell populations to suggest a leukemic/lymphomatous process. These findings are compatible with HHV-8 negative CD. The numerous polytypic plasma cells with interfollicular distribution associated and increased vascularity suggest a mixed histological subtype. The lymph node histopathology in conjunction with clinical features was suggestive of severe iMCD-TAFRO (Table 2). This diagnosis was further supported by elevated VEGF (198 pg/mL) and IL-6 (48 pg/mL, reference range <6.4 pg/mL).

**Table 2.** Summary of patient characteristics and clinical course.

Characteristic	Result
Age (years)	31
Sex	Female
Admission sCr (mg/dL)	1.1
Peak sCr (mg/dL)	4.4
Need for dialysis	Yes
Platelet nadir (X 10^9^/L)	23
Anasarca	Yes
Organomegaly	Yes
Fever or CRP > 2 mg/dL	Fever and CRP = 22.5 mg/dL
VEGF level (pg/mL)^a^	198
Respond to siltuximab	Yes after 1^st^ dose
Post-treatment sCr (mg/dL)	0.8

^a^VEGF reference range < 91 pg/mL.

CRP, c-reactive protein; IL-6, interleukin-6; sCr, serum creatinine; VEGF, vascular endothelial growth factor.

Weekly infusions of siltuximab 11 mg/kg with high-dose steroids and sulfamethoxazole/trimethoprim for *Pneumocystis jirovecii* pneumonia prophylaxis were started. After the first siltuximab dose, there were immediate improvements in kidney and respiratory functions, recovery of platelet count (120 x 10^9^/L) and a reduction in CRP (5.7 mg/dL). Kidney replacement therapy was stopped after the second dose of siltuximab, with normalization of CRP (0.5 mg/dL), platelet count (197 x 10^9^/L), and full recovery of kidney function (sCr 0.9 mg/dL). The patient was discharged with siltuximab titrated down to every 3 weeks plus a steroid taper. Prior to the third siltuximab infusion, the patient developed a mild, pruritic rash for which sulfamethoxazole/trimethoprim was switched to dapsone. Given the persistent rash, siltuximab was switched to tocilizumab, which led to resolution.

### 4.2. Case discussion

Evidence describing the kidney histopathology in iMCD-TAFRO is limited [21,25–29]. Most patients diagnosed with iMCD-TAFRO have demonstrated a pattern consistent with MPGN and TMA, which corresponds to the histopathological findings from this case report. The presence of MPGN and TMA on kidney biopsy may further increase suspicion when evaluating patients for iMCD-TAFRO. We also noted elevated VEGF levels in our case, which is in accordance with the literature for iMCD-TAFRO (Table 2). VEGF triggers the endothelial cell damage cascade (from vascular hyperpermeability and fluid leakage to anasarca) that is regularly noted in these patients [3,25,27]. Patients with iMCD-TAFRO often have normal to mildly elevated levels of IL-6 as well as increased VEGF [1,16,42]. VEGF overproduction activates the mammalian target of rapamycin (mTOR) and may drive TMA in iMCD. Moreover, it is postulated that TMA occurs in the acute phase and MPGN in the chronic phase. When examining patients for possible iMCD-TAFRO, elevated VEGF levels may be another useful diagnostic tool.

Our case supports the CDCN and NCCN Guidelines^®^, which recommend siltuximab as a first-line therapy with continued treatment until disease progression [24,38]. Weekly siltuximab was initiated upon clinical suspicion of severe iMCD-TAFRO with positive responses to treatment. While there are no prospective studies comparing treatment outcomes in patients with iMCD due to the challenges of conducting trials in rare diseases, there are real-world data to assist clinical decision making. Recent real-world data from the ACCELERATE registry cohort included 65 patients who met clinical criteria for iMCD-TAFRO as confirmed by an expert panel. These patients received a median (interquartile ratio) of 3 (2–4) treatment regimens, most commonly anti-IL-6 therapies ± steroids (*n* = 39), including siltuximab (*n* = 28) and tocilizumab (*n* = 15). Investigators found higher durable response rates (defined as ≥50% improvement in symptoms after treatment initiation lasting ≥1 year) in patients who received siltuximab ± steroids (45.8%) or tocilizumab ± steroids (35.7%) vs other treatment alternatives, particularly rituximab ± steroids (27.3%). In addition, none (0%) of the 26 patients who received corticosteroid monotherapy achieved durable response [12]. These findings reinforce the role of anti-IL-6 therapies in the management of patients with iMCD-TAFRO, with nearly half of evaluable patients achieving sustained clinical benefit. While rituximab may be effective in select cases, the rate of durable response was lower when compared to anti-IL-6 therapies. Steroid monotherapy has a high treatment failure rate in iMCD-TAFRO, but steroids may be combined with other therapies for symptom control. Of note, the real-world analysis also found a strong association between patients being diagnosed with iMCD-TAFRO and having severe disease, as observed in our case [12].

**Figure 3.**
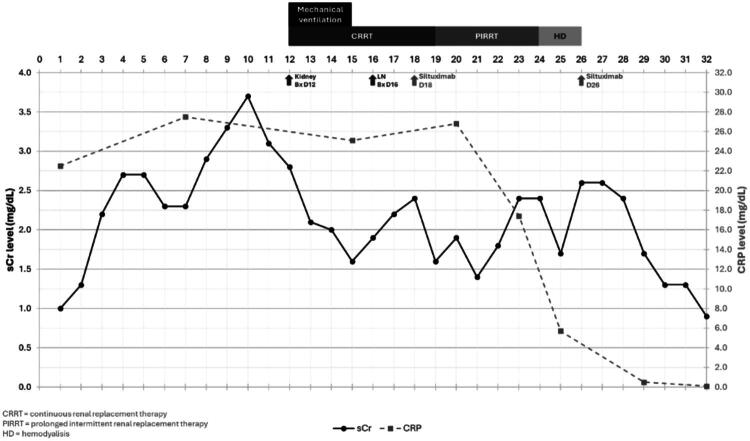
Timeline of sCr and CRP Levels and Clinical Course. Bx, biopsy; CRP, C-reactive protein; CRRT, continuous renal replacement therapy; D, day; HD, hemodialysis; LN, lymph node; PIRRT, prolonged intermittent renal replacement therapy; sCr, serum creatinine.

Our patient received weekly siltuximab with positive response, aligning with the consensus recommendation to treat severe iMCD with weekly siltuximab 11 mg/kg dosing (instead of the standard dosing of 11 mg/kg every 3 weeks) and high-dose steroids as soon as possible [24,38,43]. The CDCN guidelines recommend treating aggressively and considering therapy adjustments as early as 1 week after initiation if the patient is not responding [24]. However, it can take months for signs and symptoms to normalize in an acute setting. An analysis of siltuximab responders from the pivotal phase two study showed that labs and symptoms normalize first (usually within months 1 and 2, respectively), with delayed lymph node response (median time to lymph node response was 4.1 months) [44]. This is in line with findings from a previous real-world retrospective analysis and demonstrates the importance of regular patient monitoring, focusing on improving lab trends first, followed by symptom and lymph node response [45]. Our results reiterate the importance of monitoring labs over the course of treatment to evaluate response and make treatment adjustments as needed. This was demonstrated in our case, where frequent lab monitoring to assess trends in CRP, platelets, and acute symptoms (e.g., respiratory and kidney function) was helpful to evaluate treatment outcomes (Figure 3).

This case illustrates the successful management of rare aggressive disease with mixed signs and symptoms that add challenge in diagnosis. The patient had a full recovery of respiratory and kidney functions, which could be attributed to early recognition of the disease and successful switch between different lines of treatment. Follow-up visits will allow the team to monitor the patient’s response. Study limitations include the small sample size (*n* = 1).

In conclusion, many patients with iMCD-TAFRO present with abnormal kidney function and will require nephrology care. Our results support a pattern of renal involvement with MPGN and TMA on kidney biopsy. These findings should be factored in when evaluating patients for iMCD-TAFRO. We also highlight the importance of following diagnostic criteria and treatment guidelines to recognize and treat this rare condition as early as possible and mitigate the risk of patient decompensation.

## Supplementary Material

CARE checklist_13FEB26.docx

## Data Availability

Data sharing is not applicable to this article as no new data were created or analyzed in this study.
