# Supplementary material for: Successful treatment of idiopathic multicentric Castleman disease with TAFRO and kidney involvement: case report and literature review
Source: Ren Fail. 2026 Jun 17;48(1):2671463. doi: 10.1080/0886022X.2026.2671463 (PMC13288712; doi:10.1080/0886022X.2026.2671463)
Supplement: CARE checklist_13FEB26.docx [file IRNF_A_2671463_SM7838.docx]

The CARE reporting checklist

For checking that clinical case report articles can be understood and used by everyone

|  | Item Description | Location (or reason for not reporting) |
| --- | --- | --- |
| **Sections** |  |  |
| [1. Title](https:/resources.equator-network.org/reporting-guidelines/care/items/title.html?utm_source=care&utm_medium=checklist&utm_campaign=CARE_2013_v1_1) | The area of focus and “case report” should appear in the title. | Title, page 1. |
| [2. Keywords](https:/resources.equator-network.org/reporting-guidelines/care/items/keywords.html?utm_source=care&utm_medium=checklist&utm_campaign=CARE_2013_v1_1) | The key elements of this case in 2–5 words. | Keywords, page 2, last paragraph. |
| [3. Abstract](https:/resources.equator-network.org/reporting-guidelines/care/items/abstract.html?utm_source=care&utm_medium=checklist&utm_campaign=CARE_2013_v1_1) | 3a – Introduction: What does this case add?  3b – Case presentation:   - The main symptoms of the patient(s). - The main clinical findings. - The main diagnoses and interventions. - The main outcomes.   3c – Conclusion: What are the main “take-away” lessons from this case? | The abstract follows a free format because the current publication is primarily meant to be a literature review focused on the renal histopathology and mechanisms of kidney injury in iMCD-TAFRO. The case is included as an example of acute kidney failure observed in this condition. |
| [4. Introduction](https:/resources.equator-network.org/reporting-guidelines/care/items/introduction.html?utm_source=care&utm_medium=checklist&utm_campaign=CARE_2013_v1_1) | Brief background summary of the case referencing the relevant medical literature. | The current manuscript is meant to be published as a review; an introduction and key topics are discussed at length in pages 3-12. |
| [5a. Patient information](https:/resources.equator-network.org/reporting-guidelines/care/items/patient-information.html?utm_source=care&utm_medium=checklist&utm_campaign=CARE_2013_v1_1) | 5a – Demographic information of the patient (age, gender, ethnicity, occupation).  5b – Main symptoms of the patient (chief complaint).  5c – Medical, family, and psychosocial history—including lifestyle and genetic information whenever possible, details about relevant comorbidities, and past interv… | Age, gender, ethnicity, and medical history: Page 10, Section 4.1, Paragraph 1, line 1.  Main symptoms: Page 10, Section 4.1, Paragraph 1, lines1-5. |
| [6. Clinical findings](https:/resources.equator-network.org/reporting-guidelines/care/items/clinical-findings.html?utm_source=care&utm_medium=checklist&utm_campaign=CARE_2013_v1_1) | Describe the relevant physical examination (PE) findings. | Page 10, Section 4.1, Paragraph 1, lines1-5. |
| [7. Timeline](https:/resources.equator-network.org/reporting-guidelines/care/items/timeline.html?utm_source=care&utm_medium=checklist&utm_campaign=CARE_2013_v1_1) | Depict important date and times in this case (table or figure). | Figure 3 |
| [8. Diagnostic assessment](https:/resources.equator-network.org/reporting-guidelines/care/items/diagnostic-assessment-and-diagnosis.html?utm_source=care&utm_medium=checklist&utm_campaign=CARE_2013_v1_1) | 8a – Diagnostic methods (e.g., physical examination, laboratory testing, imaging, questionnaires)  8b – Diagnostic challenges (e.g., financial, language, or cultural)  8c – Diagnostic reasoning including other diagnoses considered  8d – Prognostic characteristics (e.g., staging) where applicable. | Page 10, Paragraph 1  Page 11, Paragraphs 1-3  Page 12, Paragraph 1  Table 2 |
| [9. Therapeutic Intervention](https:/resources.equator-network.org/reporting-guidelines/care/items/therapeutic-interventions.html?utm_source=care&utm_medium=checklist&utm_campaign=CARE_2013_v1_1) | 9a – Types of intervention (e.g., pharmacologic, surgical, preventive, self-care)  9b – Administration (e.g., dosage, strength, duration)  9c – Changes in intervention (with rationale). | Page 13, Paragraph 1  Figure 3 |
| [10. Follow up and outcomes](https:/resources.equator-network.org/reporting-guidelines/care/items/follow-up-and-outcomes.html?utm_source=care&utm_medium=checklist&utm_campaign=CARE_2013_v1_1) | 10a – Clinician and patient-assessed outcomes  10b – Important follow-up test results (positive and negative)  10c – Intervention adherence and tolerability (and how this was assessed)  10d – Adverse and unanticipated events. | Page 13, Paragraph 1  Figure 3 |
| [11. Discussion](https:/resources.equator-network.org/reporting-guidelines/care/items/discussion.html?utm_source=care&utm_medium=checklist&utm_campaign=CARE_2013_v1_1) | Discussion (including conclusion):  11a – Strengths and limitations of the management of this case  11b – Relevant medical literature  11c – Rationale for conclusions (including assessment of cause and effect)  11d – Main “take-away” lessons of this case report. | Page 13, Section 4.2  Page 14  Page 15, Paragraphs 1 and 2 |
| [12. Patient perspective](https:/resources.equator-network.org/reporting-guidelines/care/items/patient-perspective.html?utm_source=care&utm_medium=checklist&utm_campaign=CARE_2013_v1_1) | When appropriate patients should share their perspectives on the treatments they received. | Patient is willing to provide feedback upon request. |
| [13. Informed consent](https:/resources.equator-network.org/reporting-guidelines/care/items/informed-consent.html?utm_source=care&utm_medium=checklist&utm_campaign=CARE_2013_v1_1) | Did the patient give informed consent? Please provide if requested. | Yes. Page 16, Ethical approval, last sentence. |
